# Biochar improves the performance of *Avena sativa* L. grown in gasoline-polluted soils

**DOI:** 10.1007/s11356-022-24127-w

**Published:** 2022-11-19

**Authors:** Riccardo Fedeli, Dmitriy Alexandrov, Silvia Celletti, Elvira Nafikova, Stefano Loppi

**Affiliations:** 1 Department of Life Sciences, University of Siena, 53100 Siena, Italy; 2 Ufa State Aviation Technical University, Karla Marksa Str., 12, 450000 Ufa, Russia; 3 BAT Center - Interuniversity Center for Studies On Bioinspired Agro-Environmental Technology, University of Naples ‘Federico II’, 80138 Naples, Italy

**Keywords:** Bioremediation, Contamination, Oat, Petroleum, Petroleum derivatives, Phytotoxicity

## Abstract

This study investigated the effect of soil contamination by different concentrations of gasoline on oat (*Avena sativa* L.) and tested the effect of biochar supply to the polluted soils on the performance of oat plants. Oat seeds were sowed in contaminated soils with different concentrations of gasoline: 0% (control), 1%, 2%, 6%, and 10% (v/w), and grown for 2 weeks. Germination, fresh weight, root and stem length, photosynthetic parameters (i.e., chlorophyll content, PI_ABS_, F_V_/F_M_, and NDVI), and total antioxidant power were analyzed. The results showed a remarkable negative effect on almost all the investigated parameters starting from the gasoline concentration of 6%. Based on these results, a new experiment was run by adding 5% (w/w) biochar (a carbon-rich byproduct of wood biomass pyrolysis) to the 6% and 10% polluted soils to test whether adding biochar had a beneficial effect on oat performance. The results showed that biochar supply greatly reduced the negative effects caused by gasoline on all the investigated parameters.

## Introduction

Soil contamination by petroleum and its derivatives such as diesel and gasoline is a recent environmental concern which has also dramatic financial implications, especially given the extensive use and transportation of petroleum (Nazari Heris et al. 2020). Petroleum is made up of many hydrocarbons, including various heteroatomic compounds containing sulfur, oxygen, nitrogen, and metals (Speight 1998), and is harmful to plants and cannot be decomposed by native soil microorganisms (Zhang et al. 2013).

The main causes of environmental contamination from petroleum and its derivatives are related to building engineering problems (piping systems), which allow the transport of these substances from the production area to the sites of use (Doherty and Otitoloju, 2013). Although both environmental and operational variables are taken into account during the construction and installation of the pipelines, it happens that the pipelines over time, the movements of the ground, the changes in pressure and temperature, and accidental damages can break and consequently cause the spillage of the products transported along them (Sanches et al. 2013).

Recently, several environmental disasters occurred due to the rupture of oil product pipelines: in 2016 in France, an accidental rupture caused a pipeline breakage, leading to a spillage of more than 550,000 L of diesel (Life gate 2016). More recently, in January 2022, in North America due to the corrosion of a pipeline, more than 1 M L of diesel was spilled (U.S. News 2022); a month later, in Ecuador a massive rock fell on a pipeline and caused it to rupture, spilling it on land (Reuters 2020).

Similarly to petroleum, gasoline, being one of its byproducts, is composed of a complex mixture of organic substances, principally alkanes and cycloalkanes and monoaromates, with a molar weight ranging from 44 g mol^−1^ (propane, C_3_) to 142 g mol^−1^ (decane, C_10_) (Trapp et al. 2001), and if released can highly pollute the environment (Watts et al. 2000). Soil contamination by gasoline can lead to oxygen and water deficit (Anon, 2003), as well as deficiency of available forms of phosphorus and nitrogen (Wyszkowska and Kucharski 2000), thereby causing toxic effects on plants (Anon 2003; Odjegba and Sadiq 2002; Trapp et al. 2001).

Several bioremediation techniques have been applied to limit the problem of soil contamination by petroleum and its derivatives. Among them are phytoremediation techniques (Schnoor 1997; Frick et al. 1999) as well as biostimulation and bioaugmentation of microorganisms to degrade pollutants (Rajapaksha et al. 2016). Recently, a further bioremediation technique has been investigated, involving the application of biochar, a bio-based material, to decontaminate soils from diesel (Saeed et al. 2021).

Biochar is a solid carbon-rich byproduct of pyrolysis (Hagemann et al. 2018; International Biochar Association 2018), and has recently been included among soil improvers that can be used in agriculture (Legislative Decree 75 2010). The main feedstock for biochar production is plant biomass from scrap of secondary forest cutting (Yargicoglu et al. 2015) or agricultural waste (Lugato et al. 2013). Biochar can counteract various environmental problems since it increases carbon sequestration and reduces the emissions of greenhouse gases (Gupta et al. 2020). It also improves soil structure (i.e., porosity and aeration) (He et al. 2016). It is now well known that biochar can immobilize organic and inorganic substances, thus reducing the availability of toxic elements to plants and other organisms in contaminated soil (Zheng et al. 2013; Park et al. 2013; Oliveira et al. 2017; Vannini et al. 2021). Biochar can also improve crop productivity due to its nutrient content, retention of soil nutrients, increased soil cation exchange capacity, improved soil physical properties, and increased soil water retention (Laird et al. 2010).

Oat (*Avena sativa* L.) is an important cereal crop in the developing world, and it ranks sixth in world grain production statistics (Stevens et al. 2004). Oat is mainly cultivated in Russia, Canada, the USA, and Europe (Leff et al. 2004). Recently, the use of oat as animal feed has steadily declined due to the emergence of interest in its use as a human health food (Ahmad et al. 2010). Indeed, its grains are a rich source of dietary fiber, antioxidants, minerals, and vitamins (Allwood et al. 2021). This crop plant is extremely versatile, growing under a wide array of environmental conditions, including cool and humid climates as well as poorly fertile or arid areas (Buerstmayr et al. 2007), and compared to wheat or maize has a much lower nutrient demand (Rasane et al. 2015).

This study was undertaken with the aims of (1) testing the effect of soil contamination by different concentrations of gasoline on oat and (2) testing if the addition of biochar to polluted soils has a beneficial effect on the performance of oat.

## Materials and methods

### Gasoline treatments and plant growth conditions

Experimental microcosms (24 × 18 × 5 cm) were prepared using a commercial growing medium (VigorPlant Italia Srl—professional mix) kindly provided by the Botanical Garden of the University of Siena (Italy). Each microcosm was filled with 200 g of substrate and treated with increasing concentrations of gasoline to obtain the following conditions (v/w): 0% (G0, control), 1% (G1), 2% (G2), 6% (G6), and 10% (G10). These concentrations were chosen on the basis of results obtained on soils accidentally contaminated by gasoline spills (Khosravi et al., 2013; Nasehi et al. 2016a, b; Yazdi and Sharifi Teshnizi 2021). In each microcosm, 25 seeds of oat (*Avena sativa* L.), kindly provided by the Botanical Garden of the University of Siena (Italy), were then allowed to germinate. Microcosms were stored in a climatic growth chamber with 60% RH, light intensity of 300 μmol m^−2^ s^−1^ PAR at leaf level, a day/night cycle of 14/10 h, and 24/16 °C. Plants were watered when necessary to maintain soil at 70% of water holding capacity to ensure constant moisture. The experiment lasted 2 weeks.

#### Biochar addition

In a separate trial, based on the outcomes of the previous experiment, it was decided to test the addition of biochar only for the G0 (control), G6, and G10 conditions (namely B0, B6, and B10, respectively). Microcosms were prepared as described above plus 5% (w/w) biochar (type “*green sand*” branded BioDea© (BioDea 2022), kindly provided by BioEsperia Srl, Umbertide, PG, Italy. The physicochemical characteristics of biochar are listed in Table 1. Microcosms were treated with the same conditions as above.

**Table 1:** Physicochemical characteristics of biochar (type “*green sand*”, BioDea©)

Particle diameter (µm)	< 200
Total nitrogen (%)	< 0.4
Total potassium (mg kg^−1^)	3020
Total phosphorous (mg kg^−1^)	340
Total calcium (mg kg^−1^)	9920
Total magnesium (mg kg^−1^)	852
Total sodium (mg kg^−1^)	291
Carbon from carbonate (%)	< 0.1
Total carbon (%)	68.7
Water holding capacity (%)	23.5
Salinity (mS m^−1^)	110
pH	9.9
Hash content (%)	4.6
H/C	0.2

### Growing medium analysis

The pH and electrical conductivity (EC) were measured in the aqueous extracts of the growing substrate where oat plants were grown, using a pH meter (edge blu, HANNA Instruments Srl, Woonsocket, USA) and a conductivity meter (EC-meter, Delta Ohm, HD/8706, Padova, Italy), respectively. The extracts were obtained as described in Celletti et al. (2021).

### Plant analysis

#### Seed germination

The germination percentage (GP) of oat seeds was calculated starting from the 1st day after sowing until the day of harvesting to obtain a germination rate (GR) (ISTA 1999):

$$\mathrm{GP}\;\left(\%\right)=\left[\left(\frac{\mathrm{seeds}\;\mathrm{germinated}}{\mathrm{total}\;\mathrm{seeds}\;\mathrm{sown}}\right)\times100\right]$$

#### Photosynthetic parameters

The total chlorophyll content was measured using a non-destructive chlorophyll content meter (CCM-300, Opti-Science, Hudson, USA). For each plant, measurements were taken at the apical parts, avoiding leaf nerves (Fedeli et al. 2022a). The results are expressed on a surface basis (mg m^−2^).

After a 15-min dark adaptation, leaves were hit for 1 s with a beam of saturating red light at 650 nm of 2400 μmol m^−2^ s^−1^ intensity and the fluorescence emitted from the leaf was measured by using a plant efficiency analyzer (Handy PEA, Hansatech Ltd., Norfolk, UK). As photosynthetic efficiency indicators, F_V_/F_M_, the maximum quantum yield of PSII (Strasser et al. 2000), and the performance index (PI_ABS_), which is an index of plant vitality, were used.

Foliar reflectance was expressed through the normalized difference vegetation index (NDVI), which is a measure of the health status of the leaves based on a normalized ratio of the NIR (near-infrared) and red bands, and was measured using a PlantPen NDVI (Photon Systems Instruments, Czech Republic).

#### Plant fresh weight

Immediately after harvest, each plant was weighed using a precision balance. Before weighing, all plants were cleaned with a small paintbrush to get rid of the substrate. Results are expressed as grams of fresh plant weight (g).

#### Stem and root length

After weighing, the length of shoots and roots was measured with a ruler. Root length was measured from the main apex to the crown, and shoot length was measured from the crown to the main apex (Morelos-Moreno et al. 2019; Ogbo et al. 2009). Results are expressed in millimeters (mm).

#### Shoot total antioxidant power

Total antioxidant power was measured in shoots according to the method reported by Loppi et al. (2021) and Fedeli et al. (2022b) with slight modification. Briefly, approximately 100 mg of frozen oat shoots (ca.) was homogenized in 1 mL of 80% (v/v) ethanol. Subsequently, the homogenates were centrifuged at 4000 rpm for 5 min and 100 µL of each supernatant was added to 1 mL of 2,2-diphenyl-1-picrylhydrazyl (DPPH) solution prepared as follows: 3.9 mg of DPPH was dissolved in 100 mL of 80% (v/v) methanol. A blank and a control were prepared by adding the same amount of 100 µL of 80% (v/v) ethanol in 1 mL of 80% (v/v) methanol and in 1 mL of DPPH solution, respectively. The reaction occurred by keeping all the tubes in darkness for 1 h. Afterwards, the absorbance was read at the wavelength of 517 nm by means of a UV–Vis spectrophotometer (Agilent 8453, Santa Clara, CA, USA). The results were expressed as the percentage of antiradical activity (ARA, %), according to the following formula:

$$\mathrm{ARA} \left(\%\right)=\left\{100\times \left[1-\left(\frac{\mathrm{sample}}{\mathrm{control}}\right)\right]\right\}$$

### Statistical analysis

Due to the limited data set, nonparametric statistics were used. Differences (*p* < 0.05) between the effects of different gasoline and biochar treatments were checked with Kruskal–Wallis ANOVA using the Conover-Imam (1979) test for post hoc comparisons, correcting for multiple testing according to Benjamini and Hochberg (1995). Results are presented as median ± error, with the latter expressed as interquartile range divided by the square root of the number of observations. All calculations were run using the R software (R Core Team 2022).

## Results

### Effect of gasoline

Contamination with gasoline did not cause any change in substrate pH, but substrate electrical conductivity was significantly increased compared to control after treatment with 6% (+ 7.8%) and especially 10% (+ 9.4%) gasoline (Table 2).

**Table 2:** pH and EC (µS cm^−1^) of the substrate used for the growth of oat plants. Values are expressed as median ± error. *G *= without biochar; *B* = with 5% (w/w) biochar. Different letters (lowercase for G, capital for B) indicate statistically significant (*p* < 0.05) differences between treatments

Treatment	Gasoline (%)	pH_soil 1:20 (w/w)_	EC_soil 1:20(w/w)_
G	0	7.05 ± 0.10^a^	1212 ± 21^c^
B		7.32 ± 0.06^A^	1177 ± 36^A^
G	1	7.24 ± 0.016^a^	1217 ± 42^c^
B		-	-
G	2	7.27 ± 0.09^a^	1231 ± 41^c^
B		-	-
G	6	7.23 ± 0.03^a^	1306 ± 30^b^
B		7.51 ± 0.01^A^	1100 ± 18^A^
G	10	7.22 ± 0.01^a^	1325 ± 39^a^
B		7.59 ± 0.09^A^	1159 ± 30^A^

**Fig. 1:**
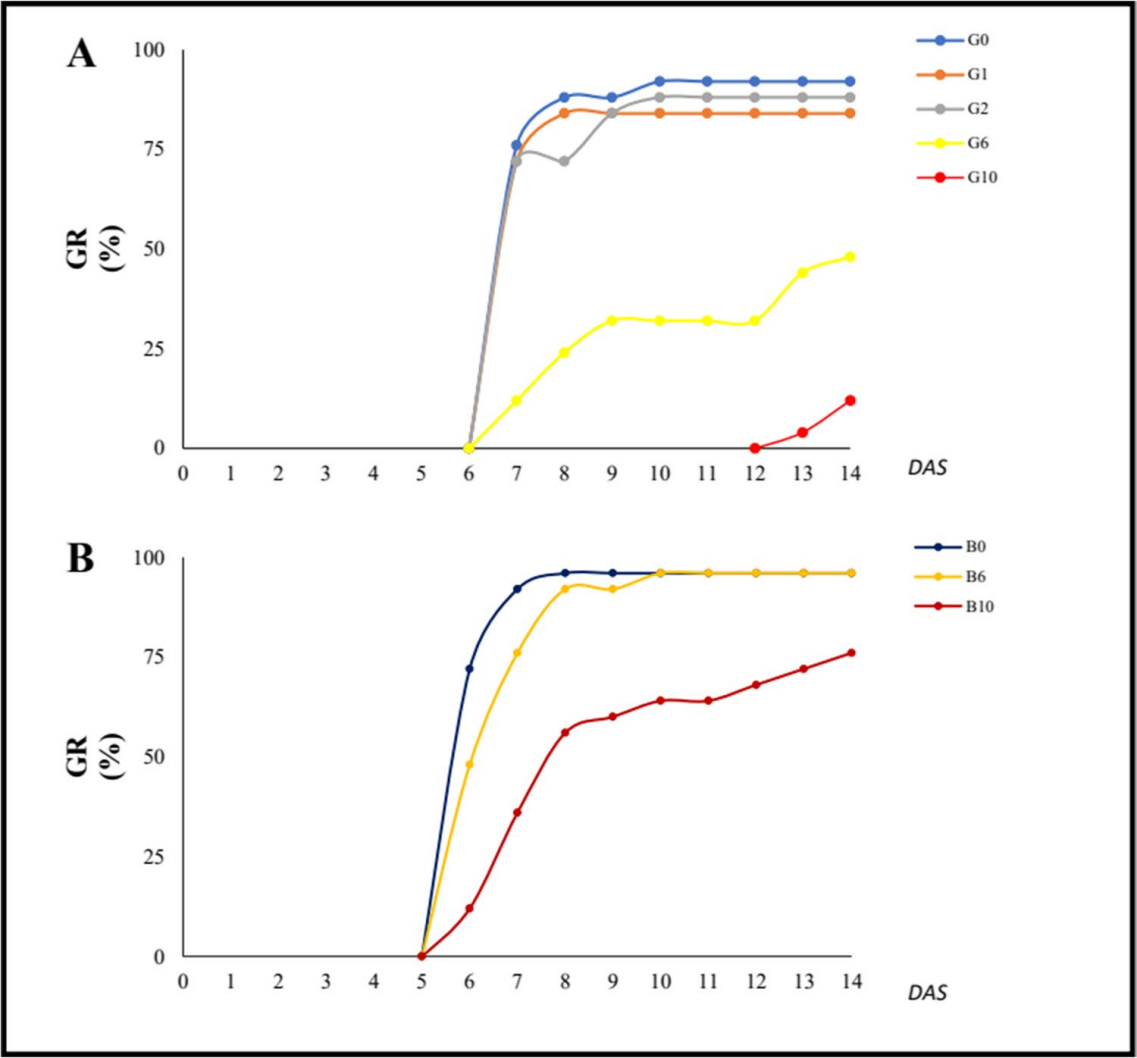
Germination rate (GR) of oat seeds without (**A**) and with (**B**) the addition of 5% (w/w) biochar to the substrate. **G** = without biochar; **B** = with 5% (w/w) biochar. DAS, days after sowing

The germination rate of oat seeds sowed in 1% and 2% gasoline added to the growing substrate was similar to the control, while a remarkable decrease in seed germination was observed at 6% (− 47.8%) and especially at 10% (− 90%) addition of gasoline (Fig. 1).

**Fig. 2:**
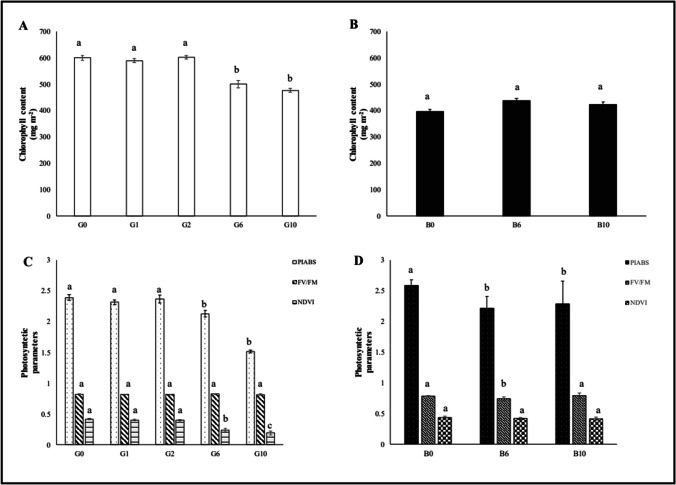
Chlorophyll content and photosynthetic parameters (PI_ABS_, F_V_/F_M_, NDVI) measured in oat leaves (median ± error). **A** and **C:** G = without biochar. **B** and **D:** B = with 5% (w/w) biochar. Different letters indicate statistically significant (*p* < 0.05) differences between treatments

At physiological level, a significant reduction of leaf chlorophyll, PI_ABS_, and NDVI was evident, but only at 6% and 10% gasoline addition to the growing substrate (Fig. 2). The decreases compared to the control were as follows: content of chlorophyll: − 43.9% and − 54.2%, PI_ABS_: − 16.6% and − 20.6%, NDVI: − 11.0% and − 36.7%, at G6 and G10 conditions, respectively. The parameter F_V_/F_M_ did not show any statistically significant difference with the control at all gasoline concentrations tested.

**Fig. 3:**
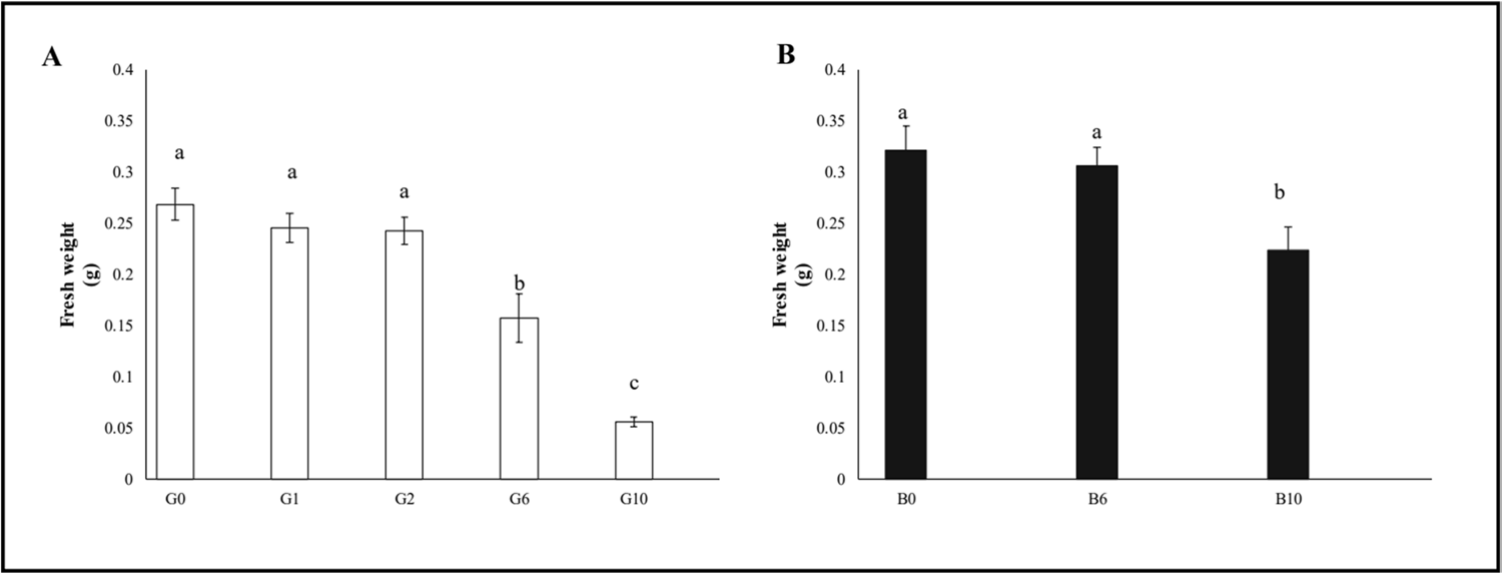
Fresh weight of oat plants (median ± error). Number along the horizontal axes indicates the percentage of gasoline in the soil. Different letters indicate statistically significant (*p* < 0.05) differences between treatments. **A:** G = without biochar. **B:** B = with 5% (w/w) biochar

A similar decreasing trend was observed for the fresh weight of the samples (Fig. 3), with conditions G1 and G2 not showing significant differences with the control, and a marked decline at G6 (− 41.2%) and especially G10 (− 79.1%).

**Fig. 4:**
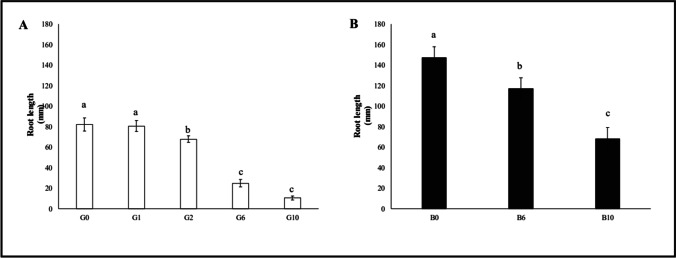
Root length of oat plants (median ± error). Number along the horizontal axes indicates the percentage of gasoline in the soil. Different letters indicate statistically significant (*p* < 0.05) differences between treatments. **A:** G = without biochar. **B:** B = with 5% (w/w) biochar

**Fig. 5:**
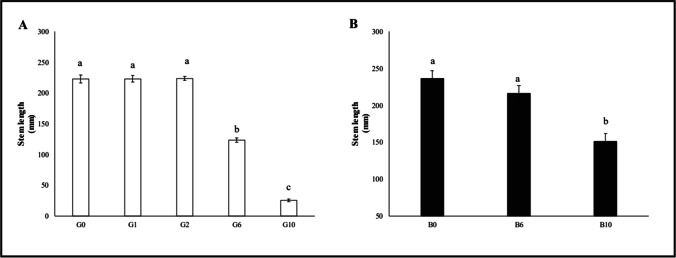
Stem length of oat plants (median ± error). Number along the horizontal axes indicates the percentage of gasoline in the soil. Different letters indicate statistically significant (*p* < 0.05) differences between treatments. **A:** G = without biochar. **B:** B = with 5% (w/w) biochar

Again, a reduction of stem length compared to control was measured only in G6 (− 44.6%) and G10 (− 88.6%) samples (Fig. 4). The length of the root system (Fig. 5) was reduced already at G2 (− 17.5%), and the reduction markedly continued at G6 (− 69.8%) and G10 (− 87.2%).

A significant reduction in the total antioxidant power was evident in the G6 shoots (− 64.2%). Unfortunately, it was not possible to perform this analysis for the G10 condition since, owing to the detrimental effect of gasoline on oat plants, the raw material was not sufficient.

### Effect of gasoline and biochar

With the addition of 5% biochar to the growing substrate, gasoline contamination did not influence substrate pH and EC (Table 2).

A significant decreased germination of oat seeds was observed only at B10 (− 20.8%) compared to the control (Fig. 1).

The photosynthetic parameters of oat leaves did not show any statistically significant difference with the controls (Fig. 2).

As far as plant fresh weight is concerned (Fig. 3), a significant reduction was observed only at B10 (− 20.83%).

Stem length (Fig. 4) showed a significant reduction only at B10 (− 36.03%), while the length of the root system (Fig. 5) was significantly reduction both at B6 (− 20.55%) and especially B10 (− 53.58%).

**Fig. 6:**
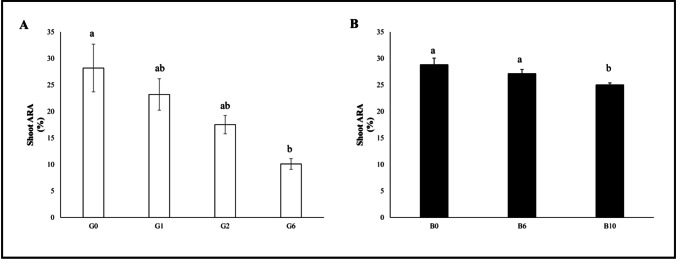
Shoot antioxidant power (ARA%) of oat plants (median ± error). Number along the horizontal axes indicates the percentage of gasoline in the soil. Different letters indicate statistically significant (*p* < 0.05) differences between treatments. **A:** G = without biochar. **B:** B = with 5% (w/w) biochar

The total antioxidant power was decreased (− 13.2%) only in the B10 samples (Fig. 6).

### Effect of biochar supply

To properly compare the results obtained without and with biochar addition to the substrate, the values obtained for the 6% and 10% gasoline contamination were expressed as ratios to the respective controls (Table 3).

**Table 3:** Treated to control ratios (median ± error) of each parameter at 6% and 10% soil gasoline contamination. *G*, without biochar; *B*, with 5% (w/w) biochar. *Statistically significant (*p* < 0.05) difference between treatments at the same gasoline concentration

	G		B	
	6	10	6	10
pH	1.03 ± 0.01	1.02 ± 0.01	1.03 ± 0.01	1.04 ± 0.01
EC (µS cm^−1^)	1.08 ± 0.03	1.09 ± 0.03	0.94 ± 0.02*	0.99 ± 0.03*
Fresh weight (g)	0.59 ± 0.09	0.21 ± 0.02	0.95 ± 0.05*	0.70 ± 0.07*
Root length (mm)	0.30 ± 0.04	0.13 ± 0.02	0.80 ± 0.07*	0.46 ± 0.06*
Stem length (mm)	0.55 ± 0.09	0.05 ± 0.01	0.92 ± 0.03*	0.64 ± 0.05*
Chlorophyll content (mg m^−2^)	0.83 ± 0.03	0.79 ± 0.08	1.10 ± 0.02*	1.06 ± 0.03*
PI_ABS_	0.89 ± 0.03	0.63 ± 0.09	0.86 ± 0.05	0.82 ± 0.04*
F_V_/F_M_	1.00 ± 0.01	0.99 ± 0.01	0.97 ± 0.03	1.00 ± 0.02
NDVI	0.59 ± 0.07	0.46 ± 0.03	0.96 ± 0.04*	0.95 ± 0.06*
Shoot ARA (%)	0.36 ± 0.06	-	0.94 ± 0.03*	0.87 ± 0.05

As far as the growing substrate is concerned, there was no evidence of significant change in pH, while EC showed a decrease with the addition of biochar at both concentrations tested: − 13.3% at B6 and − 9.9% at B10.

The germination rate of oat seeds increased both at B6 (+ 92.3%) and especially at B10 (+ 507.7%).

The chlorophyll content showed a significant increase at B6 (+ 32.1%) and B10 (+ 34.0%). NDVI was a significantly increased at both B6 (+ 71.0%) and B10 (+ 107.6%). PI_ABS_ was significantly increased only at B10 (+ 39.9%), while F_V_/F_M_ did not show any significant difference.

A significant increase in plant fresh weight was observed for both the conditions: B6 (+ 62.1%) and especially B10 (+ 232.8%).

Stem length was increased at B6 (65.2%) and especially B10 (+ 240.3%); similarly, root length was increased at B6 (+ 163.1%) and also at B10 (+ 9.7%).

The total antioxidant power also showed a marked increase at B6 (+ 163.2%); since it was not possible to run this analysis on the G10 samples, a direct comparison is not feasible for B10.

## Discussion

### Oat response to gasoline contamination

Our results clearly showed that a 6% gasoline soil contamination is sufficient to hinder the growth and the development of oat seedlings, consistently with several studies in the literature. Adetitun et al. (2013) observed inhibitory effects on the germination of green amaranth (*Amaranthus viridis* L.) plants from concentration 6% gasoline in the soil, while these effects were not observed at lower concentrations (1% and 3%). Adam and Duncan (2002) treated soils with 2.5% and 5% diesel and sowed seeds of red fescue (*Festuca rubra* L.), silky fescue (*Festuca ovina* L.), laurel (*Agropyron repens* L.), ryegrass (*Lolium multiflorum* L.), and common hay (*Poa trivialis* L.); after 2 weeks, while the lowest diesel concentration did not show any effect, the highest concentration totally hampered the germination. The same findings were obtained by Dib and Sadoudi Ali Ahmed (2020) on common cocklebur (*Xanthium strumarium* L.) seedlings, with no adverse effect on germination upon 2.5% diesel addition to soil, while starting from a concentration of 5% up to 10%, a decrease in the germination rate was found. The same trend was observed when petroleum was added instead of its derivatives. As an example, 15 days after sowing, common hay plants grown in soils with 1% and 2% petroleum did not show any significant germination difference compared with the control, while, from 5%, the germination rate decreased reaching the minimum values at 10% and 15% petroleum concentrations (Minai-Tehrani 2008). All the above evidence strongly suggests that there is a tolerance threshold at about 5% that plants have toward hydrocarbons in the soil, beyond which there is clear evidence of toxic effects by petroleum and its derivatives on plant growth.

The aboveground biomass is generally correlated with a longer length of stems, while the belowground biomass accumulation with a greater elongation of the root system (Sun et al. 2015; Enquist and Niklas 2002; Shipley and Meziane 2002). Our results indeed showed that as gasoline concentration in the substrate increased, stem and root length and fresh weight of oat seedlings decreased; we can speculate that this is probably caused by the reduction of nitrogen and phosphorus availability due to the capacity of petroleum and its derivatives to reduce their availability (Baran et al. 2002; Agbogidi et al. 2007). Our results are in agreement with several studies. Fatokun and Zharare (2015) studied the effects of 0.1–30% diesel supply in the soil on the growth of lettuce (*Lactuca sativa* L.) and sweet potato (*Ipomoea batatas* L.) plants, and Etukudo et al. (2011) studied the effects of 1–5% diesel contamination of soils on the growth of okra (*Abelmoschus esculentus* L. Moench) plants, and both studies showed a notable drop in biomass and in the length of both stems and roots from the concentration of 5%. The same deleterious impact was observed when increasing concentrations (up to 15%) of petroleum were supplemented in the soils where both common hay and gray mangrove (*Avicennia marina* L.) plants were grown, leading to a significant decrease in both root and stem length (Moradi et al. 2020) as well as in plant biomass (Minai-Tehrani 2008).

Usually, a reduction in plant growth and development is caused by a reduction in plant photosynthetic efficiency. Prominently, our findings highlighted a decline in photosynthesis-related parameters, such as chlorophyll content, PI_ABS_, and NDVI, assayed in leaves of oat plants grown in soils treated with 6% and 10% gasoline. The observed decrease in chlorophyll content is consistent with several studies showing similar reductions due to soil pollution with petroleum and its derivatives (Baker 1970; Njoku et al. 2009). Moreover, Achuba and Iserhienrhien (2018) showed that the chlorophyll content in cowpea (*Vigna unguiculata* L.) leaves decreased as gasoline concentrations (from 0.10 to 2%) added to the soil increased. Similarly, Ezeonu and Onwurah (2009) and Odjegba and Sadiq (2002) observed the same negative effect on chlorophyll content in maize (*Zea mays* L.) by contaminating the soil with petroleum, and on amaranthus (*Amaranthus hybridus* L.) plants by contaminating the soil with exhausted engine fuel. It is known that PI_ABS_ is an excellent indicator of the damage induced by both abiotic and biotic stress on the photosynthetic apparatus (Maxwell and Johnson 2000), with low PI_ABS_ values indicating low vitality and difficulty to assimilate carbon (Kumar et al. 2020). Our results showed that PI_ABS_ decreased as the concentration of gasoline in the substrate increased. Such reduction is consistent with previous studies reporting a toxic effect of petroleum on maize plants exposed to concentrations of 2.5% and 5% added to the soil (Athar et al. 2016; Tomar et al. 2015; Tomar and Jajoo 2013).

The total antioxidant power in the shoots of oat plants was investigated to assess the intrinsic defense response of oat to the oxidative damage caused by the presence of phytotoxic compounds in gasoline added to the soils where the plants were cultivated. The potential of this analysis relies in the fact that it provides an estimation of the total pool of different classes of antioxidant substances, including flavones, isoflavones, flavonoids, anthocyanins, coumarin, lignans, catechins, and isocatechins (Aqil et al. 2006). In plants as well as in animals, by counteracting the negative action of reactive oxygen species or free radicals, the antioxidant pool limits the oxidative stress and, thereby, inhibits cell membrane lipid peroxidation and protects living organisms against potential cellular damage (Gupta and Sinha 2009). A high antioxidant level is thus an indication of prevention of lipid peroxidation and, therefore, of oxidative stress damage at the expense of plants (Chakravarty and Deka 2021). Our results are consistent with those of Rusin et al. (2018), which showed that the presence of petroleum and its derivatives in the soil lowered the level of antioxidants in wheat (*Triticum aestivum* L.) leaves.

### Responses following biochar supply to gasoline-polluted substrate

Several techniques have been proposed for remediating contamination from petroleum and its derivatives, e.g., addition of surfactants, microbial activities (Liu et al. 2021; Huang et al. 2019). Recently, the use of biochar as an eco-friendly product for bioremediation of diesel-contaminated soils has been investigated for the first time by Saeed et al. (2021). Owing to its capacity to limit plant uptake of organic and inorganic contaminants from polluted soils (Lu et al. 2015; Oliveira et al. 2017; Vannini et al. 2021), biochar may be effectively used for the bioremediation of soils contaminated by petroleum and fuels, but so far there is a lack of information on this aspect. It is widely reported in the literature (e.g., Blanco-Canqui 2019; Laird et al. 2010) that biochar has a high capacity to retain water, so keeping the water content constant across treatments was necessary to avoid introducing an additional variable that could have influenced the results. Obviously, in field reality, it is impossible to have soils constantly at a certain water content. Therefore, it would be of great interest to study the influence that different water contents could have on the capability of biochar to limit the damage caused by gasoline on crops. This is the first study reporting that the addition of 5% biochar can counteract the negative effects caused by soil contamination with petroleum derivatives. Based on our outcomes, one of the positive effects resulting from the addition of biochar to gasoline-polluted soils was the notable increase in seed germination rate. It is well known that seed germination and plant growth are mainly hampered by a low soil nitrogen availability (Walker et al. 2001), and it has been demonstrated that petroleum and its derivatives can reduce the availability of several soil nutrients such as N and phosphorus (Baran et al. 2002; Agbogidi et al. 2007). This might be the reason of the largely reduced germination rates of oat seeds we have found at 6% and 10% gasoline concentrations in the substrate. On the other hand, consistently with our results, Gul and Whalen (2016) and Zheng et al. (2013) showed that the addition of biochar can enhance the persistence of both N and P in the soil, avoiding their leaching, and, consequently, increasing their availability for plants.

Our findings are in line with those of Saeed et al. (2021), where the use of biochar for bioremediation of diesel-contaminated soils was investigated in a pot experiment with the addition of 10% and 15% diesel fuel into the soil with and without the addition of 1% (w/w) biochar. Overall, a positive effect was observed in the plants grown with biochar, particularly on the fresh weight, at both 10% (+ 25.5) and 15% (18.2%) diesel concentrations. Also, the content of chlorophyll was increased at all concentrations of diesel in the soil, with an average increase of about 10% (Saeed et al. 2021). There is also evidence that the addition of biochar significantly increased the antioxidant content of plants, as well as several other parameters, i.e., proline, total amino acids, soluble sugars, and total proteins (Chakravarty and Deka 2021). Although the mechanisms of biochar mitigation have not been approached experimentally, we can speculate that the most likely is adsorption of the phytotoxic gasoline compounds to the biochar, as also reported by Saeed et al. (2021) for diesel contamination. In addition, also increased N and P availability, as documented by Nelson et al. (2011) and Gul and Whalen (2016), could play an important role. As a first experimental study on the possible use of 5% (w/w) biochar to counter gasoline contamination, further field studies will be needed to verify the real viability of its use in this specific environmental problem. Furthermore, it will be necessary to investigate whether the response observed by the addition of biochar is across all soils and if changes observed might be due to the soil microbial component.

## Conclusions

This study showed that a minimum of 6% gasoline soil pollution has a wide array of negative effects on *Avena sativa*, an important cereal crop, now widely used as human food. Moreover, we have shown that the addition of 5% biochar to the polluted soils has a beneficial effect on the performance of oat.

The search for effective bio-based solutions for environmental issues is an urgent goal of utmost importance. Here, we suggest that biochar is a useful tool to remediate gasoline-contaminated soils, enhancing both seed germination and growth of oat plants. Nevertheless, in spite of the lack of knowledge about the use of biochar in the remediation of polluted soil by petroleum and its derivatives, additional studies are necessary to support our promising results.

## Data Availability

The raw data presented in this study are available on request from the corresponding author.
